# Patient and family experience with telemedicine and in-person pediatric and obstetric ambulatory encounters throughout 2020, during the COVID-19 epidemic: the distance effect

**DOI:** 10.1186/s12913-022-08037-8

**Published:** 2022-05-16

**Authors:** Sandro Marques, June Alisson Westarb Cruz, Maria Alexandra Viegas Cortez da Cunha, Felipe Francisco Tuon, Thyago Proença de Moraes, Alaís Daiane Zdziarski, Sean T. Bomher, Lane F. Donnelly, Robson Capasso

**Affiliations:** 1 Stanford Medicine, California, USA; 2 Pontifícia Universidade Católica Do Paraná, Curitiba, Brazil; 3 Fundação Getulio Vargas, Rio de Janeiro, Brazil; 4 Stanford Children’s Health, California, USA

**Keywords:** Telehealth, Patient Experience, Quality improvement, In-person visits, Likelihood to Recommend, Maternal and children’s hospital, Logistic regression

## Abstract

**Background:**

Telemedicine has grown significantly in recent years, mainly during the COVID-19 pandemic, and there has been a growing body of literature on the subject. Another topic that merits increased attention is differences in patient and family experience between telehealth and in-person visits. To our team’s knowledge, this is the first study evaluating pediatric and obstetrics outpatients experience with telemedicine and in-person visit types in an academic maternal and children’s hospital, and its correlation with geographic distance from the medical center throughout 2020, during the COVID-19 crisis.

**Methods:**

We aim to evaluate and compare patients’ telemedicine and in-person experience for ambulatory encounters based on survey data throughout 2020, during the COVID-19 pandemic, with particular focus on the influence of distance of the patient’s home address from the medical facility. A total of 9,322 patient experience surveys from ambulatory encounters (6,362 in-person and 2,960 telemedicine), in a maternal and children’s hospital during 2020 were included in this study. The percentage of patients who scored the question “Likelihood to recommend practice” with a maximum 5/5 (top box) score was used to evaluate patient experience. The k-means model was used to create distance clusters, and statistical t-tests were conducted to compare mean distances and Top Box values between telemedicine and in-person models. Logistic regression analysis was used to evaluate the correlation between Top Box scores and patients’ distance to the hospital.

**Results:**

Top Box likelihood to recommend percentages for in-person and telemedicine were comparable (in-person = 81.21%, telemedicine = 81.70%, *p*-value = 0.5624). Mean distance from the hospital was greater for telemedicine compared to in-person patients (in-person = 48.89 miles, telemedicine = 61.23 miles, *p*-value < 0.01). Patients who live farther displayed higher satisfaction scores regardless of the visit type (*p*-value < 0.01).

**Conclusions:**

There is a direct relationship between the family experience and the distance from the considered medical center, during year 2020, i.e., patients who live farther from the hospital record higher Top Box proportion for “Likelihood to Recommend” than patients who live closer to the medical center, regardless of the approach, in-person or telemedicine.

## Background

**Fig. 1:**
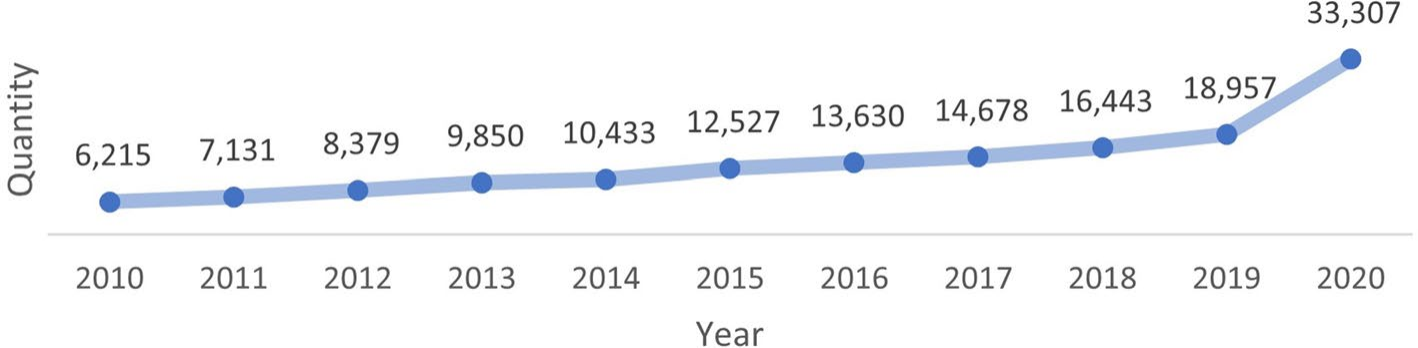
Number of telemedicine-related publications between 2010 and 2020 (PUBMED, Scopus, Web of Science, Sage, and Scielo)

The term Telemedicine is used to define the exchange and use of medical information to deliver medical services from different sites, by using electronic communication networks. [1, 2]. Although telemedicine is not a novel concept [3–5], it has grown significantly in recent years, in particular during the COVID-19 pandemic when non-face-to-face healthcare became essential [6, 7]. Consequently, there is increased interest on its outcomes and indications, and there has been a growing body of literature on the subject, as shown on Fig. 1.

**Fig. 2:**
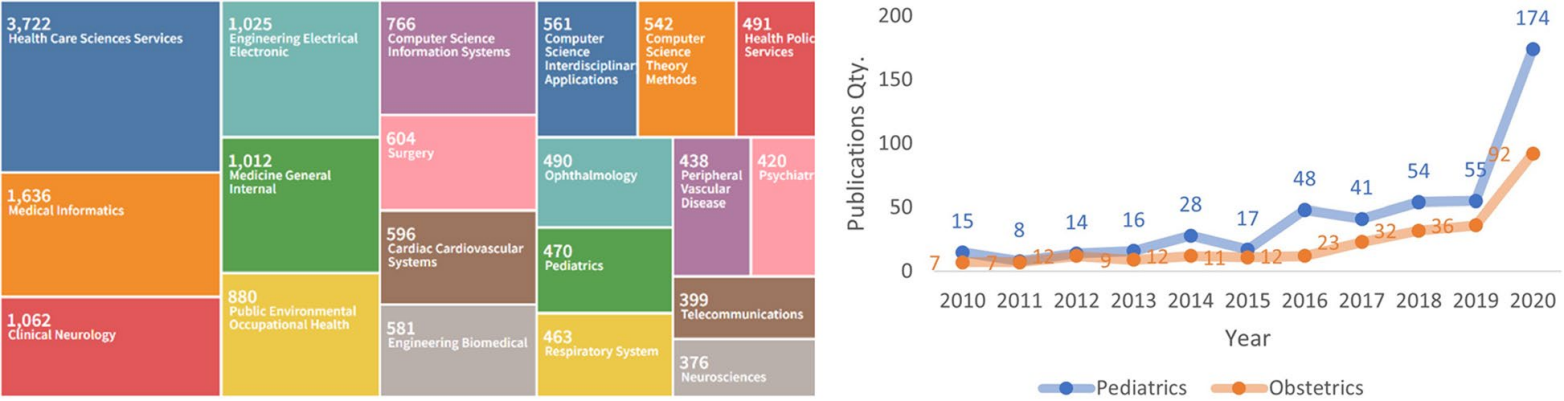
**A** Research subareas of publications in telemedicine and (**B)** quantity of publications on Pediatrics and Obstetrics (Web of Science)

Based on data obtained from the Scopus (Elsevier) and Web of Science databases, the pediatrics is one area with few publications (470), as showed at Fig. 2 A. Obstetrics has even less (253), and it is not showed at picture Fig. 2 A.

There has been an increase in publications for these two specifics area, mainly in recent years: in pediatrics, it is noted an increase from 55 publications in 2019 to 174 in 2020, while in obstetrics, it increased from 36 in 2019 to 92 in 2020 (Fig. 2 B).

Another topic that merits increased attention is data from surveys of patient and family experience, but specifically for the two specialties considered here, there are just publications about telemedicine and patient experience. While the ideal methodological approach to measure it is still a matter of discussion [8, 9], proxies such as patients’ experience survey scores are still the most commonly utilized tools. Literature reviews and development of frameworks for telemedicine satisfaction measurements have been proposed [10, 11]. However, to our team’s knowledge, this is the first study evaluating pediatric and obstetrics outpatients experience with telemedicine and in-person visit types in a large US-based academic medical center, and its correlation with geographic distance from the medical center during the COVID-19 crisis.

## Methods

### Study design and population

This is a retrospective analysis of patient’s experience survey data administered at a maternal and children’s hospital and associated clinics. Questionnaires were collected from January to December 2020 for in-person and telehealth visit types. Data included primary care and subspecialized pediatric and obstetric outpatient services.

As part of our institutional oversight of quality, safety, and service – patient experience survey scores are monitored to determine where we are improving and what targeted actions may need to be taken. Following our institutional guidelines, this project met criteria as quality improvement activity and was not considered human subjects research, and as a result did not require approval by our Institutional Review Board.

### Data collection

The 16-question patient experience survey was electronically collected from patients and families who underwent to medical services, in-person or via telehealth. The same 16-question patient experience survey (Table 1) was used in other studies developed at this hospital unit: “The survey is a tailored version of an ambulatory survey created in cooperation with a vendor (Press Ganey, South Bend, IN), validated and used at multiple healthcare systems throughout the United States” [12].

**Table 1:** Questionnaire used in the evaluation of patient or guardian experience following face-to-face or telemedicine medical care

#	Question
1	Care provider's discussion of any proposed treatment (options, risks, benefits, etc.)
2	Care provider's efforts to include you in decisions about your treatment
3	Concern the care provider showed for your questions or worries
4	Concern the nurse/assistant showed for your problem
5	Courtesy of staff in the registration area
6	Degree to which you were informed about any delays
7	Ease of contacting (e.g., email, phone, web portal) the clinic
8	Ease of scheduling your appointment
9	Explanations the care provider gave you about your problem or condition
10	How well staff protected your safety (by washing hands, wearing gloves, etc.)
11	How well staff worked together to care for you
12	How well the nurse/assistant listened to you
13	Likelihood of your recommending our practice to others
14	Likelihood of your recommending this care provider to others
15	Our concern for your privacy
16	Wait time at clinic (from arriving to leaving)

Each question was evaluated on a scale of 1–5. A grade of five (5/5) was considered *Top Box* [12]*.* The organization considers the percentage of *Top box* scores as a standard metric for patient experience.

For the purposes of this study, the answers to the question 13—*Likelihood of your recommending our practice to others* (LTR) were considered for further analysis. This metric has been extensively studied, and is frequently utilized in patient experience improvement efforts [13–15].

Patient’s distance to the medical facility was calculated by an interface with Google Maps service utilizing the zip code for each patient’s residence. The Top Box data was then stratified by micro-regions, and the averages were weighted by the number of responses from each zip code.

### Statistical analysis

Zip codes were clustered according to distance to the medical center, and a k-means model, a clustering algorithm that associates an identification to each group of elements that share the same cluster, was used [16].

The distance between the elements and each centroid is calculated using a cartesian plane with clusters on the *x*-axis and distances on the *y*-axis. The number of centroids defines the number of desired clusters. If only one centroid is considered, we will have only one cluster. With two or more defined centroids, each element is associated with the centroid closest to it. Since the number of centroids (or clusters) desired must be indicated in the algorithm, a calculation was made to determine the ideal number of clusters to minimize the sum of the distances of each element to the centroids. In a range from 1 to 15 centroids, the amount that minimizes this sum and, therefore, the ideal number of clusters, was three [3].

As all patients living within the same zip code were considered to have the same distance to hospital, distance outliers were eliminated. To compare mean values for Top Box and distance for telehealth and in-person groups, a statistical t-test was conducted. To evaluate the relationship between distance and visit type (independent variables) with Top Box (dependent variable), a logistic regression model was used. All statistical analysis was made using R Studio [17].

## Results

A total of 9,322 patient experience surveys (for 6,362 in-person and 2,960 telemedicine encounters) were analyzed, from pediatric and obstetrics outpatients encounters during year 2020. They were answered by patients or families living in 30 American states, 406 cities, and 694 zip codes.

Table 2 shows that most micro-regions were located less than 193.77 miles from the center, with a mean of 248.16 and a median of 93.44 miles. This big difference between mean and median is influenced by 126 individual patient surveys, which addresses have the farthest 93 zip codes, with distance greater than 421.61 miles (limit value to be statistically considered an outlier) and although few, significantly influenced the means. Therefore, we excluded the evaluations from these 93 zip codes as they were considered outliers, leaving 9,196 individual patient encounters. Therefore, 98.6% of the patient encounters and 86.6% of the original zip codes were retained in the study.

**Table 2:** Descriptive analysis of the distance variable for micro-regions (Zip codes)

			**Variable Zip code Distance (miles)**					
**Database**	**Evaluation Qty**	**Zip code Qty**	**Min**	**1st Qu**	**Median**	**Mean**	**3rd Qu**	**Max**
All data	9,322	694	0.0	42.35	93.44	248.16	193.77	3,105.71
No outliers	9,196	601	0.0	39.40	82.66	109.78	160.95	419.31
**%**	**98.6%**	**86.6%**						

**Fig. 3:**
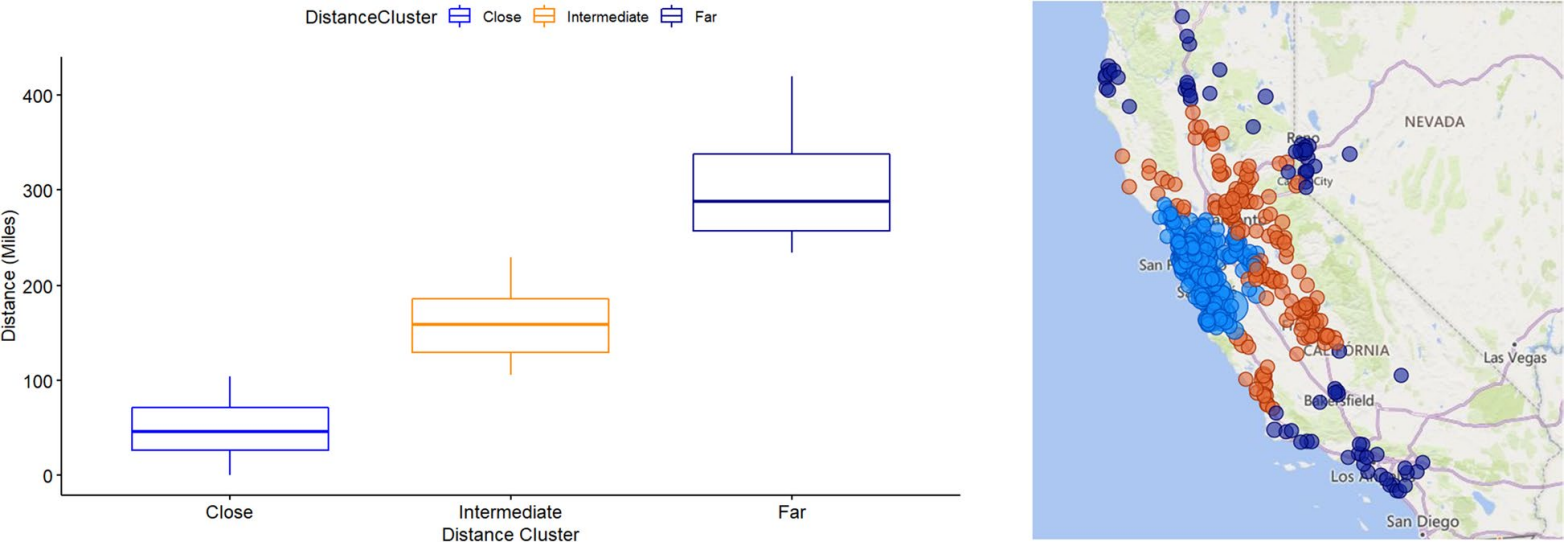
Distance clusters and map representation

For clarity, clusters were renamed to represent the average distance from the cluster to the hospital as "Close," "Intermediate," and "Far". According to their distances to the hospital, the cluster compositions are presented in Fig. 3, which is a representation of zip codes and clusters. For “Close”, the median distance from the zip code to the hospital was 46.1 with a range of 0.0 to 104.3 miles. For “Intermediate”, the median distance from the zip code to the hospital was 159.0 with a range of 105.7 to 229.3 miles. For “Far”, the median distance from the zip code to the hospital was 288.0, with a range of 233.7 to 419.3 miles.

**Fig. 4:**
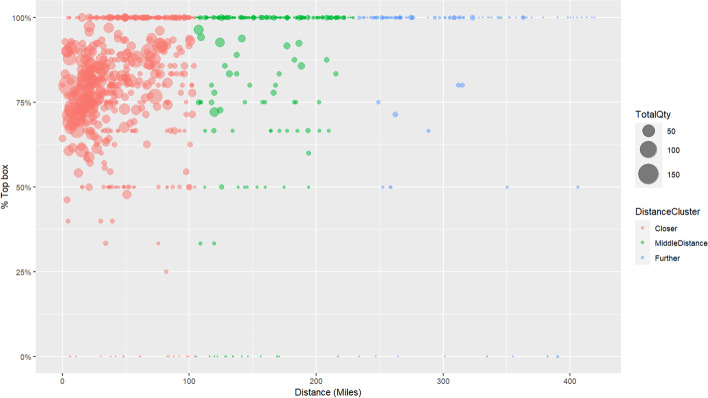
Scatter plot of zip codes—Top box * Distance * Quantity

The distribution of zip codes and the associated mean score for “likelihood to recommend” are presented in Fig. 4, where each zip code is represented by a circle, and the size of the circle represents the number of evaluations in that micro-region. Note that most experience scores in the “Close” cluster are above the value of 50% for Top Box LTR.

**Fig. 5:**
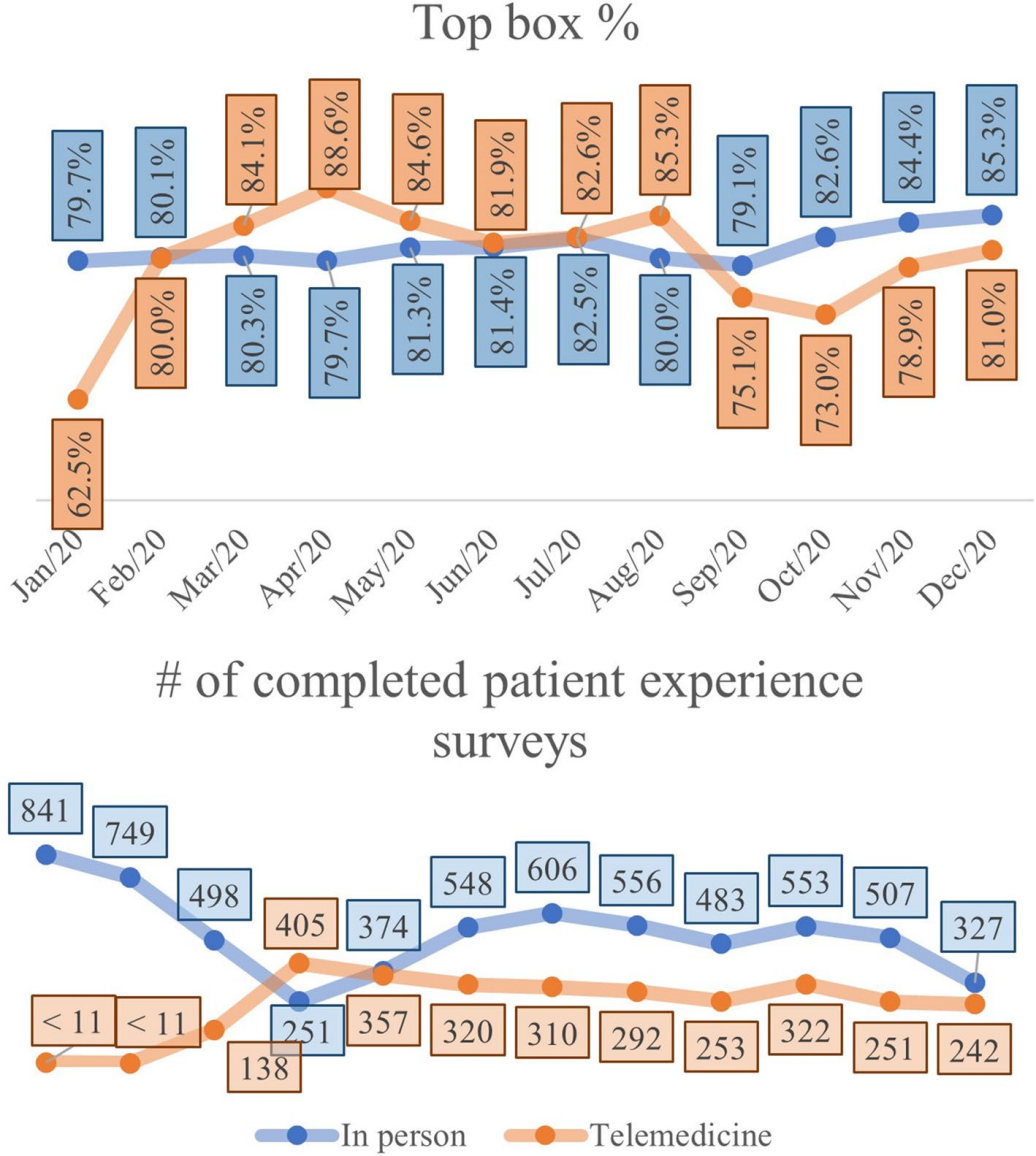
Top box (1A) and number of completed patient experience surveys (1B) per month by visit type

Average patient experience by visit type (in-person or telemedicine), and number of completed surveys are shown in Fig. 5. Despite monthly variations in the Top Box percentages for each modality, the t-test comparing the mean Top Box between two visit types, showed that there was no statistical difference between the average of telemedicine and in-person service evaluations (in-person = 81.21%, telemedicine = 81.70%, *p*-value = 0.5624) for the time period of calendar year 2020.

### Average patient distance by visit type

**Fig. 6:**
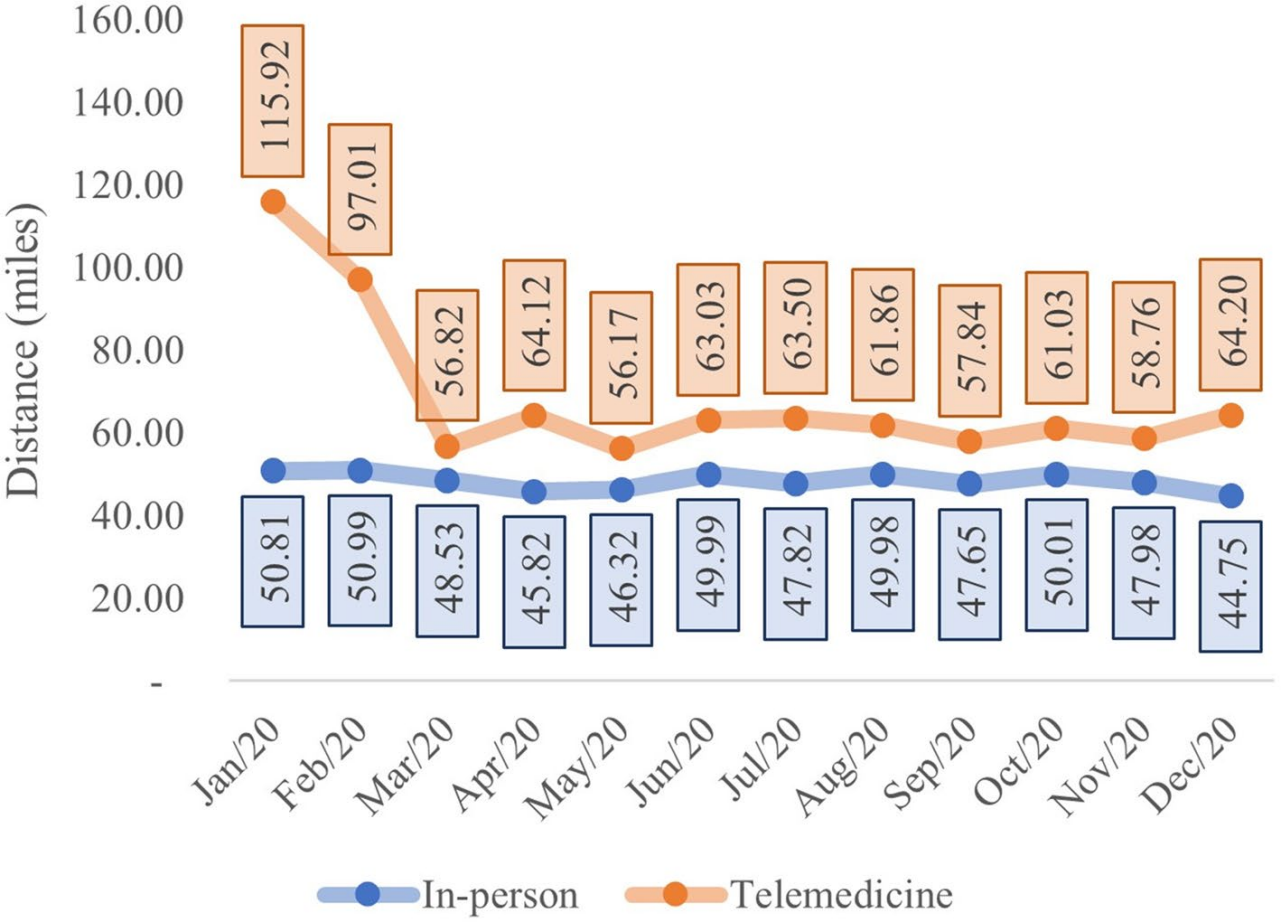
Average distance (miles) per month and in-person vs telehealth visit type

The relationship between average distance from the patient’s home and the healthcare center by visit type is shown in Fig. 6.

Comparing the average distance of consultations by telemedicine relative to in-person, t-test analyses demonstrate a statistical difference (in-person = 48.90 miles, telemedicine = 61.24 miles, *p*-value < 0.01) confirming that the average distance of telemedicine services was greater than average distance of patients on in-person visit type, as shown in Table 3.

**Table 3:** Descriptive analysis of variable distance in the evaluations by in-person vs telehealth visit type

		**Variable Zip code Distance (miles)**					
**Visit Type**	**Evaluation Qty**	**Min**	**1st Qu**	**Median**	**Mean**	**3rd Qu**	**Max**
In-person	6,293	0.0	17.48	30.75	48.90	67.41	417.06
Telemedicine	2,903	0.0	19.14	34.28	61.24	76.81	419.31

### Patient top box evaluation by distance and visit type

**Fig. 7:**
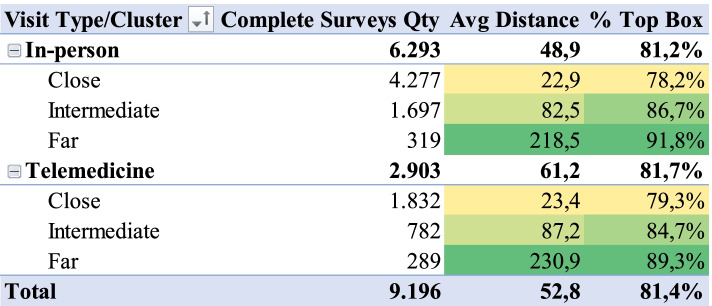
- Top box vs. visit type and distance cluster

Considering that distance is a relevant factor for telemedicine (the prefix ‘tele’, from the Greek ‘telos’, implies distance [18]), we analyzed the relation between visit type, distance and Top Box proportion for LTR for patient surveys during 2020. The variable Top Box was created from transformation of variable LTR: if the LTR was equal to 5, Top Box value was defined to 1; if the LTR was less than 5, the Top Box value was defined to 0. Figure 7 shows that there is a statistically significant relation between average patient’s distance from hospital and Top Box proportion for LTR. The greater the average distance, the greater the proportion of Top Box. This trend is valid for both telemedicine and in-person services.

The Top Box proportion for the “Close” cluster was 78.2% for in-person and 79.3% for telemedicine. For the “Intermediate” it was 86.7% for in-person and 84.7% for telemedicine, and for the “Far” cluster, the proportion of Top Box was 91.8% for in-person and 89.3% for telemedicine.

Logistic regression confirmed that there was a statistically significant difference between the Top Box proportion, used as dependent variable, and patient distance and service type considered as the independent variables, all considering 95% confidence interval. This result was found for regression using individual evaluations where Top Box was the dependent variable and patient distance was the independent variable (OR [1.0065; 1.0083] and *p*-value < 0.01). The residual analysis confirmed a well-adjusted model. This is the same result for regression using cluster Top Box proportion as dependent and cluster average distance as the independent variable: *p*-value < 0.01 for "Intermediate" (OR [2.5471; 3.8600]) and *p*-value < 0.01 for "Far" (OR [1.6794; 1.9731]) considering "Close" as the baseline.

In our geographic region, location is highly related to median household income [23]. Our healthcare center is located in the middle of Silicon Valley in which many zip codes have high median household incomes. Patients who travel farther distances to our medical center often come from the Central Valley (Fig. 3). Many of the zip codes in this region have a lower median household income. It is possible that patients and families from areas with lower median household incomes are more appreciative and provide higher patient experience scores. This is speculative but one of the limitations to our evaluation of differences Further studies are required on this topic.

## Conclusion

In conclusion, our analysis revealed a direct relationship between the proportion of Top Box evaluations, as a proxy for patient and family experience with pediatric and obstetric services, and the distance from the considered medical center, during year 2020, i.e., patients who live farther from the hospital record higher Top Box proportion for “Likelihood to Recommend” than patients who live closer to the medical center, regardless of the approach, in-person, or telemedicine. The possible reason for this direct relationship was not evaluated and could be analyzed in future research.

## Data Availability

The datasets generated and/or analyzed during the current study are not publicly available due to Stanford Medicine's institutional policies, as quality/performance data access is limited, but are available from the corresponding author on reasonable request.
